# A Fatal Case of Vomiting in the Ninth Month of Pregnancy

**Published:** 1855-11

**Authors:** H. K. Pusey

**Affiliations:** Garnettsville, Ky.


MEDICINE AND SURGERY.

NOVEMBER, 1855. NEW SERIES. VOL. IV.—NO. 5

### ART. I.—A FATAL CASE OF VOMITING IN THE NINTH MONTH OF PREGNANCY.

BY H. K. PUSEY, M. D., OF GARNETTSVILLE, KY.

VOMITING during pregnancy is of such frequent occurrence that it is regarded as one of the strongest presumptive signs of that condition. It usually occurs in the first months of pregnancy, the attack generally coming on in the morning, but it is not always confined to these periods; for it may occur at any time during the term of utero-gestation, or at any hour during the day or night. After the first and second months it occurs most frequently, perhaps, in the eighth and ninth months; when, in addition to the reflex action of the nervous system, which induces it in the early stage, encroachment of the enlarged uterus upon the abdominal organs acts as an exciting cause.

Vomiting in pregnancy is not always as harmless a symptom as the indifferent manner in which it is usually regarded might lead one to suppose. It sometimes constitutes a formidable malady. DUBOIS speaks of having met with twenty fatal cases of vomiting in pregnancy. There are also a number of other similar cases on record. Professor STOLTZ states that three out of four cases under his observation proved fatal.

On the 27th of May last I was called to see Mrs. A., aged about twenty-three years, recently from Mississippi, eight and a half months advanced in pregnancy with her second child. She told me she had suffered with distressing acidity of the *primæ viæ* during her whole pregnancy, for which she had taken large quantities of magnesia, chalk, etc. The acidity and burning of the stomach became more intolerable as pregnancy advanced, and was attended with occasional vomiting and sickness. On the day previous to my visit she was attacked with vomiting which had been almost incessant through the day and night, alternated with intensely burning eructations. The tongue was coated, bowels constipated, very slight epigastric tenderness, pulse 100 per minute, and feeble, surface natural.

The treatment employed in this case was calomel in small and repeated doses, with anodynes, alkalies, pellets of ice, counter-irritants, and all such remedies as are known usually to allay vomiting, almost all of which were rejected as promptly as they were given. Mercury produced its effects on the gums and secretions; the bowels were moved in the course of thirty-six hours by means of suppositories of soap, after repeated injections had failed; the discharges were consistent and bilious but without any abatement of vomiting.

Febrile symptoms gradually increased from day to day as did also the intolerance of the stomach. Everything that was swallowed, even to a sip of cold water or a pellet of ice, was instantly rejected. The only respite that could be obtained from this distressing symptom was by total abstinence from everything in the shape of food or drink. I soon became convinced of the gravity of the case with which I had to contend, and that the only safety for my patient consisted in premature labor, which I had determined to induce, as I thought, in time to save her. I had the operation under consideration on the sixth day of the disease, and intended to procure a consultation on the following morning; but on that evening I was summoned in great haste to attend her in labor. I found her delivered, as her mother informed me, by one single and protracted pain, of a nearly mature male child. The secundines having been expelled with the child I found the womb firmly contracted on itself. There was no hemorrhage, and she had lost but little blood in the labor. She was quite comfortable, and we were soon gratified to find the stomach quiet; she did not vomit after the expulsion of the child. At 7 o'clock, P. M., I gave her morphine to procure rest, and ordered brandy panada, and weak animal broths, which were easily retained.

On the following morning I learned that she had slept a little, though she had passed rather a restless night. I found the evidences of prostration, which before had not been at all alarming, increasing rapidly; pulse 130 per minute, vertigo and slight delirium; restless and anxious appearance of countenance; bowels had not been moved. Continued the morphine, brandy, and nourishment, with the addition of 2 grs. of quinine every three hours. I visited her several times during the day but found no amendment in her condition. She continued to sink, and died on the morning of the 3d of June, eight days from the time the vomiting had set in, and about thirty-six hours after the birth of her child.

In reference to the induction of premature labor, it seems to be according to the voice of the profession and of humanity, if indeed it involves the life of the fœtus, provided the safety of the mother imperatively demands it. But this case ran so speedily to a fatal exhaustion, and the evidences of that condition were so slight previous to labor, that I did not suspect immediate danger; hence the operation was delayed, and finally not performed because nature assumed the responsibility, and expelled the child at a time when, in my judgment, the circumstances were altogether favorable to her recovery.

*November* 3, 1855.

